# High resolution mapping of QTLs for fruit color and firmness in Amrapali/Sensation mango hybrids

**DOI:** 10.3389/fpls.2023.1135285

**Published:** 2023-06-07

**Authors:** Manish Srivastav, Nidhi Radadiya, Sridhar Ramachandra, Pawan Kumar Jayaswal, Nisha Singh, Sangeeta Singh, Ajay Kumar Mahato, Gitanjali Tandon, Ankit Gupta, Rajni Devi, Sreekanth Halli Subrayagowda, Gulshan Kumar, Pragya Prakash, Shivani Singh, Nimisha Sharma, A. Nagaraja, Abhijit Kar, Shalini Gaur Rudra, Shruti Sethi, Sarika Jaiswal, Mir Asif Iquebal, Rakesh Singh, Sanjay Kumar Singh, Nagendra Kumar Singh

**Affiliations:** ^1^ Division of Fruits and Horticultural Technology, Indian Council of Agricultural Research (ICAR)- Indian Agricultural Research Institute, New Delhi, India; ^2^ Genomics Laboratory, Indian Council of Agricultural Research (ICAR)- National Institute for Plant Biotechnology, New Delhi, India; ^3^ Division of Agricultural Bioinformatics, Indian Council of Agricultural Research (ICAR)- Indian Agricultural Statistics Research Institute, New Delhi, India; ^4^ Division of Food Science and Postharvest Technology, Indian Council of Agricultural Research (ICAR)- Indian Agricultural Research Institute, New Delhi, India; ^5^ Division of Genomic Resources, Indian Council of Agricultural Research (ICAR)- National Bureau of Plant Genetic Resources, New Delhi, India

**Keywords:** Color, firmness, fruit quality QTLs, *Mangifera indica* L., molecular linkage map, SNP markers

## Abstract

**Introduction:**

Mango (Mangifera indica L.), acclaimed as the ‘king of fruits’ in the tropical world, has historical, religious, and economic values. It is grown commercially in more than 100 countries, and fresh mango world trade accounts for ~3,200 million US dollars for the year 2020. Mango is widely cultivated in sub-tropical and tropical regions of the world, with India, China, and Thailand being the top three producers. Mango fruit is adored for its taste, color, flavor, and aroma. Fruit color and firmness are important fruit quality traits for consumer acceptance, but their genetics is poorly understood.

**Methods:**

For mapping of fruit color and firmness, mango varieties Amrapali and Sensation, having contrasting fruit quality traits, were crossed for the development of a mapping population. Ninety-two bi-parental progenies obtained from this cross were used for the construction of a high-density linkage map and identification of QTLs. Genotyping was carried out using an 80K SNP chip array.

**Results and discussion:**

Initially, we constructed two high-density linkage maps based on the segregation of female and male parents. A female map with 3,213 SNPs and male map with 1,781 SNPs were distributed on 20 linkages groups covering map lengths of 2,844.39 and 2,684.22cM, respectively. Finally, the integrated map was constructed comprised of 4,361 SNP markers distributed on 20 linkage groups, which consisted of the chromosome haploid number in Mangifera indica (n =20). The integrated genetic map covered the entire genome of Mangifera indica cv. Dashehari, with a total genetic distance of 2,982.75 cM and an average distance between markers of 0.68 cM. The length of LGs varied from 85.78 to 218.28 cM, with a mean size of 149.14 cM. Phenotyping for fruit color and firmness traits was done for two consecutive seasons. We identified important consistent QTLs for 12 out of 20 traits, with integrated genetic linkages having significant LOD scores in at least one season. Important consistent QTLs for fruit peel color are located at Chr 3 and 18, and firmness on Chr 11 and 20. The QTLs mapped in this study would be useful in the marker-assisted breeding of mango for improved efficiency.

## Introduction

Mango (*Mangifera indica* L.) belongs to the plant family *Anacardiaceae* and has historical, religious, and economic importance. It is a diploid fruit tree with 20 chromosome pairs and a small haploid genome size of ∼439 Mb (Arumuganathan and Earle, 1991). Cytogenetic analysis based on a partial allopolyploid genome for mango has also been suggested (Mukherjee, 1950). In the last two decades, enough evidence has been produced of the inheritance of genetic markers in a disomic fashion, which confirms the diploid nature of mango (Duval et al., 2005; Schnell et al., 2005; Viruel et al., 2005; Schnell et al., 2006; Singh et al., 2016; Kuhn et al., 2017).

World mango production was 57.37 million tons in 2020 from an area of 56.8 million hectares. The majority (76%) of the world production comes from Asia, followed by America (12%) and Africa (11.8%). Mango is commercially cultivated in 102 countries. India’s share in the world’s mango production is 41.6%, followed by a 10% share in China (Bally et al., 2021). In 2020, the global mango export volume was 2.3 million tons, accounting for a 3.2 billion USD export value (FAOSTAT, 2020). Indian share in the global mango export is very less accounting for <5.0% of the global trade in 2020.

Mango fruit size, firmness, color, and aroma are quality characteristics of this climacteric fruit that need to be investigated at the genomic level to improve mango fruit quality (Bally et al., 2021). These traits are important factors determining the suitability of cultivars for domestic as well as overseas markets. In general, consumers from America and Europe prefer attractive, red-colored fruits having a pleasant aroma, a blend of sweet and sour tastes, and moderate sweetness. However, consumers from Gulf countries and the Indian continent prefer very sweet aromatic mangoes. One of the major objectives in most mango breeding programs globally is the attractive peel color in hybrids, which makes the fruits more attractive and export worthy. In addition, fruit quality parameters like total soluble solids, acidity, sugars, carotenoid contents, flavor compounds, etc. are important traits that decide the superiority of a variety. Mango breeding programs targeting overseas markets consider red peel color and high fruit firmness as important traits to improve. Fruit firmness has great value in the transportation, storage, and processing of mango. However, the genetics of these traits in mango is poorly understood. Despite the recognized high quality of a few well-known mango cultivars, considerable cultivar improvement is needed in most regions of mango culture. Mangoes have a wider adaptability to both tropical and subtropical regions of the world (Singh et al., 2016). Mango originated in the South East Asian or Indo-Myanmar region, having 69 recognized species originating as forest trees with fibrous and resinous fruits (Kostermans and Bompard, 1993). Mango cultivation began at least 4,000 years ago in India (Mukherjee, 1953). The majority of commercial cultivars have originated as chance seedlings and occupied prominent places in domestic as well as overseas markets.

Although domestication and selection of mango varieties have occurred for thousands of years, the systematic breeding of mangoes is relatively recent. Mango is a difficult fruit species to handle in breeding programs due to inherent problems of long juvenility, high heterozygosity, polyembryony, and significant fruit drop, which result in low recovery of hybrid fruits. As a result, the development of meaningful mapping populations and their use in understanding the genetics of horticultural traits has been limited. In India, breeding efforts to develop mango varieties with desirable traits started seven decades ago at the ICAR-Indian Agricultural Research Institute (IARI), New Delhi, and have a notable history of varietal improvement. To date, 10 mango hybrids have been released for commercial cultivation. Mango hybrids, namely Amrapali and Mallika, identified in the 1970s, became the choice of growers at the domestic level and got the attention of mango breeders globally. Amrapali, being a highly regular, dwarf, profuse bearer with excellent fruit quality, is the preferred parent in mango breeding programs. Considering consumer demand and potential overseas markets for mild sweetness, attractive colorful peel, and pleasant aroma, the Floridian cultivar Sensation has been used as a male donor parent to impart the red peel color in Amrapali, which otherwise has light green peel at maturity. These efforts yielded several hundred full-sib bi-parental progeny populations showing phenotypic polymorphism for agronomic traits and served as a core resource for genetic studies in mango (Ramachandra et al., 2021). Full-sib hybrid populations from two known parents chosen for their horticultural traits are more effective in the construction of genetic linkage maps and are considered a powerful tool to identify linkages between traits and markers for MAS (Ogundiwin et al., 2009; Martínez-García et al., 2013; Harel-Beja et al., 2015; Kuhn et al., 2017).

Despite huge economic significance, genomic resources for mango have been limited. The first genetic map of mango produced by Kashkush et al. (2001) was with a relatively low number of markers, which limited the accuracy and resolution of the resulting linkage map. The first draft of a whole genome sequence of mango was reported in India (Singh et al., 2014; Singh et al., 2016). A high-resolution map reported by Luo et al. (2016) may prove useful in genetic studies. Kuhn et al. (2017) reported a consensus genetic map based on seven populations and significant traits-markers association. However, the marker density across 20 LGs was relatively lower. In the current decade, a wealth of information on the mango genome has been generated (Singh et al., 2014; Luo et al., 2016; Singh et al., 2016; Kuhn et al., 2017; Wang et al., 2020). This advancement in mango genomics has opened a new vista and may contribute to future mango improvement programs. Our group led by Prof. Nagendra Kumar Singh made significant progress in whole genome sequencing of mango using next-generation sequencing technologies and identified millions of SNP markers from the genome sequence data (Mahato et al., 2016; Singh et al., 2016; Iquebal et al., 2017). An increase in the number of unbiased markers and a highly resolved genetic map are essential molecular tools for mango breeders (Kuhn et al., 2017). The demand for new improved cultivars having desirable quality traits is difficult to address by breeders only relying on traditional breeding techniques. The adoption of molecular genomic tools has the potential to identify markers associated with important horticultural traits and, in general, improve the efficiency of mango breeding programs.

In the present study, we generated a high-resolution integrated genetic map based on segregation from female, male, and both parents. This map may serve as a valuable resource that can be used to improve efficiency and overcome the challenges in mango breeding. We also used the genetic linkages to study the association of SNP marker(s) with fruit color and firmness traits. We report here the identification of significant QTLs governing the color and firmness of mango fruit. The findings of this study are novel and have significance in improving the breeding efficiency of mango.

## Materials and methods

### Mapping population

**Figure 1:**
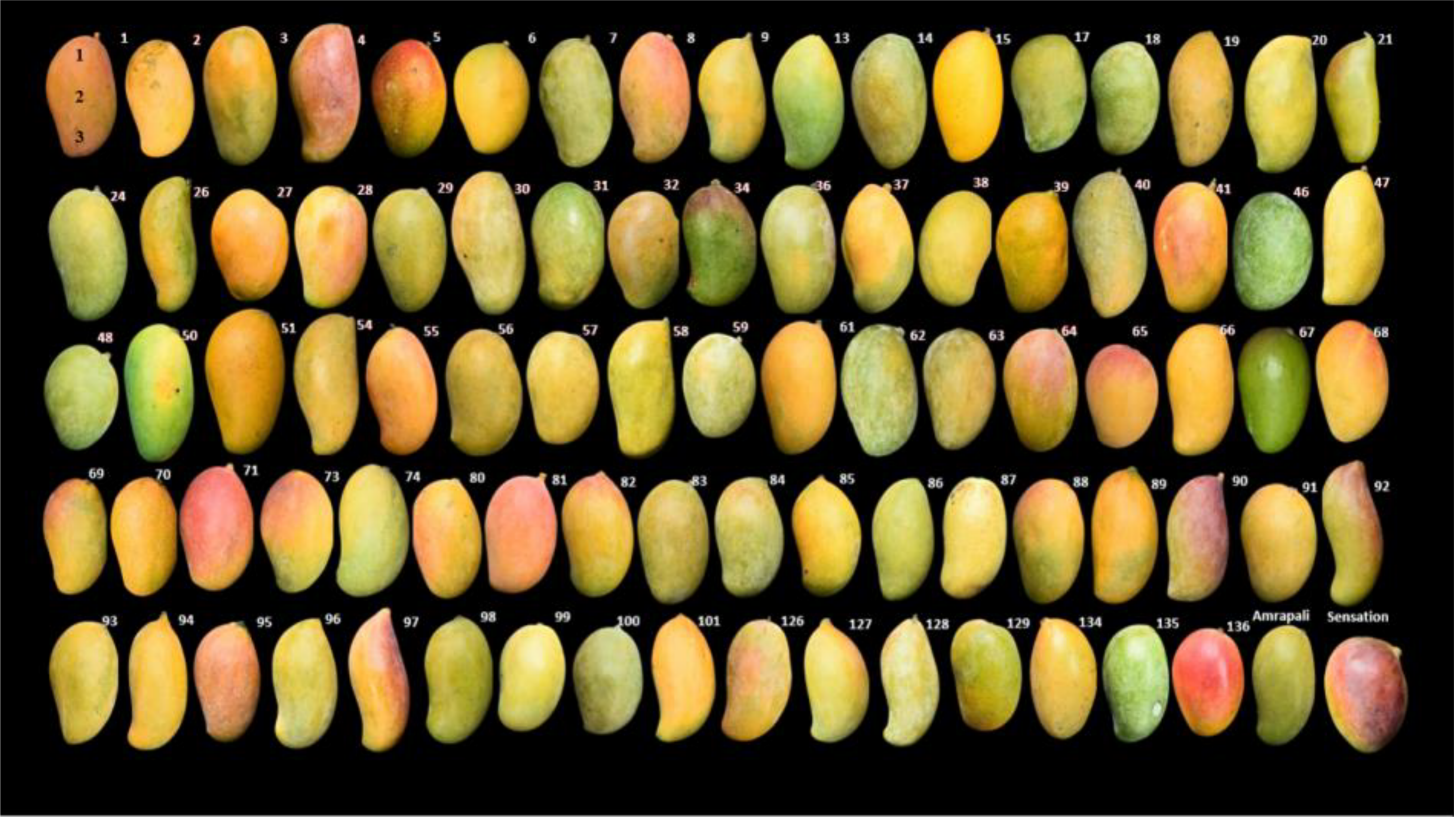
Variations in mango fruit peel color in mapping population and parents. Fruit No. 1 showing location where peel color and firmness was measured: 1. shoulder, 2. middle, and 3. bottom.

Ninety-two F_1_ bi-parental hybrids obtained from a cross of Amrapali as a female parent and Sensation as a male parent along with the parents were used for the QTL mapping. These parents were selected based on their contrasting features for key fruit quality-related traits including fruit color and firmness. The hybridity of the progenies has been confirmed earlier using SSR markers (Ramachandra et al., 2021). The population was phenotyped for multiple years and shows considerable variation for different fruit quality traits (**Figure 1**). This mapping population is conserved at the field repository of ICAR-Indian Agricultural Research Institute, New Delhi, India.

### Genotyping of the mapping population and parents

Genotyping of the mapping population and parents in duplicate was carried out using an Affymetrix mango genome 80K SNP genotyping chip array (MiSNPnks 96 array, Dr. N. K. Singh, unpublished) having a total of 18,816 genes belonging to five different categories of genes such as single-copy mango genes (SCM), conserved single-copy genes between citrus and mango (CSCCM), cloned horticulturally important mango genes (HIM), disease resistance defense response-like mango genes (DRDRM), and multi-copy genes (MCR). The average SNP density is ~6 per gene, which is highly significant in linkage mapping and QTL identification studies in mango.

DNA for genotyping was isolated from the young leaves of individual trees of the mapping population and parents following the CTAB method as modified by Ramachandra et al., 2021. Genomic DNA quality was checked by electrophoresis in 1% agarose gel and quantified using a nanodrop spectrophotometer (NanoDrop 2000 Spectrophotometer, Thermo Scientific, USA). For target probe preparation, 50 μl of genomic DNA with a concentration of 10 ng/ul was used according to Affymetrix Axiom^®^ 2.0 Assay Manual. The DNA samples were pre-amplified using Target Prep Protocol QSCB1 (P/N 702990), fragmented and hybridized on the chip, followed by single-base extension through DNA ligation and signal amplification. The Affymetrix GeneTitan^®^ platform was used for staining, washing, and scanning of the chip signals as per manufacturer’s protocol. SNP allele calling was done using Axiom™ Analysis Suite version 2.0 using its three workflows, i.e., best practices, sample QC, genotyping, and summary on the Affymetrix Gene Titan. The Axiom Analysis Suite requires stored library files to convert CEL files into genotype calls. SNPs with low call rate across the samples were removed, and only good quality SNPs with a DQC of >0.85 and call rates of >95% were used for further analysis.

### SNP data formatting

Genotype calls from all SNP markers generated by the Affymetrix GeneTitan^®^ platform from 92 progenies and parents were appended into a single csv file for export to Excel. A total of 80,816 SNPs were amplified, out of which loci with >5% missing data were filtered out. Markers amplified in only one of the two replications of the parents were also removed. The SNP allelic patterns between parents were taken as a reference for allele assignment in the mapping population. Monomorphic SNP markers were removed because they would not be informative for finding recombination events. Further, homozygous SNP markers for different alleles between the parents (aa, bb, or *vice versa*) were also removed, as there would be no segregation for such markers in the F_1_ population. Thus, a total of 32,916 informative polymorphic SNP markers were identified between Amrapali and Sensation (**Table 1**).

**Table 1:** SNP genotyping data formatting.

Type	SNPs
Total SNPs	80,816
SNPs (>95% call rate)	67,188
SNPs homozygous for same allele (aa/aa or bb/bb)	27,226
SNPs homozygous for different alleles (aa/bb or bb/aa)	7,046
SNPs heterozygous in one parent and homozygous in other or heterozygous in both parents (ab/bb or aa/ab or ab/ab)	32,916

### Linkage mapping

Based on analysis of allelic variation between parents for each polymorphic locus, SNP markers were classified as per the expected Mendelian segregation ratio in the mapping population. This set of SNP markers were mapped on the Dashehari physical map (unpublished) to assign their location on the chromosomes, and only those which mapped back on the chromosomes were retained. Polymorphic SNPs identified between parents represented multiple SNPs per gene. Therefore, only single SNPs per gene were selected (**Tables 1**, **2**).

**Table 2:** Details of polymorphic SNP markers mapped on Dashehari physical map.

Reference genome	Segregation	Allelic pattern	Expected segregation	SNPs	SNPs mapped	One SNP/gene	SNPs used for linkage mapping
Dashehari	Amrapali	ab/aa or bb	1:1	14,763	11,631	6,269	3,317
	Sensation	aa or bb/ab	1:1	10,435	8,450	4,886	2,447
	Amrapali/Sensation	ab x ab	1:2:1	7,718	6,097	3,554	1,517
				32,916	26,178	14,709	7,281

Linkage analysis was performed using homozygous SNP markers in one parent and heterozygous in the other parent (lm × ll or nn × np) and heterozygous in both parents (hk × hk) using JoinMap version 4.1 (Kyazma, Wageningen, The Netherlands). Further, the selection of SNP markers was based on their disomic inheritance and chi-square test of goodness of fit (p >0.05). Initially, for the construction of individual female and male maps, 4,834 markers in lm × ll and hk × hk, and 3,964 markers in nn × np and hk × hk were used, respectively. The initial grouping of markers was based on the independence log of the odds (LOD) tests in step ranging from 1.0 to 7.0. Other parameters were set as default. The regression mapping algorithm and Kosambi’s mapping function with minimum LOD of 3.0 were used for calculating the marker’s order. Markers showing suspect linkages were excluded in phases. Integration of female and male maps in which genotypes of some or all loci were determined in both populations took place, and the data from the separate populations were combined to calculate the integrated map. The groups that related to the same LG with at least two loci in common were combined by using the combined groups from the map integration function of the Join menu. The recombination frequencies and LOD scores of the selected sets of loci were combined into a combined group node in the navigation tree.

Such a combined group node is identical to a group node of a pairwise data population. The map calculations are based on mean recombination frequencies and combined LOD scores. For each pair of loci, the numbers of recombinant and non-recombinant gametes in the individual populations were calculated from the estimated recombination frequencies and corresponding LOD scores. The total numbers of recombinant and non-recombinant gametes of overall populations were calculated by totaling the numbers of the individual populations. From this, the mean recombination frequency and the combined LOD score were obtained. For map integration, the regression mapping algorithm was used.

### Fruit quality measurement

Matured fruits from the F_1_ trees of Amrapali/Sensation population and parents were carefully harvested with a 2 cm pedicel portion. The fruits were selected from all direction of the tree for evaluating fruit color and firmness traits for two consecutive years, 2019 and 2020. Fruits were washed thoroughly to remove the adhering dirt and dust, and rolled over the blotting paper to remove extra moisture on the surface and air dried. Fruit maturity was determined after harvest, and fruits having a specific gravity of ~1.01 to 1.02 were selected. These fruits were then wrapped in kite paper and placed in wooden boxes to ripen uniformly at room temperature. Initially, 30 fruits per individual genotype were subjected to ripening, of which 12 fruits showing uniform ripening and that were free from damage were selected for further analysis.

Peel and pulp color in terms of L*, a* and b* were measured using a Hunter-Lab Colorimeter (Model No. Miniscan^®^ XE plus 4500 L, Hunter Associates Laboratory, Inc., VA, USA). The instrument (45 / 0 geometry, D 65 optical sensor, 10 observer) was calibrated with black and white reference tiles through the tri-stimulus values X, Y and Z, taking as standard values those of the white background (X = 79.01; Y = 83.96; Z = 86.76) tile. Fruit peel color was measured at three points on the fruit surface, i.e., shoulder, middle, and bottom (**Figure 1**). Mango pulp collected from ripe fruits was homogenized, and the color of homogenized pulp was measured using a ring and disk attachment.

The firmness of ripe mango fruits was measured with the help of the TA-XT Plus Texture Analyzer (Stable Micro Systems, UK). A 2.0 mm diameter stainless steel cylinder probe was used for the test in compression mode using the load cell of 5 kg capacity. Fruit firmness was expressed in Newton (N). Firmness was measured at three points (shoulder, middle, and bottom) of the fruits of each individual and parents (**Figure 1**) with pre-test, test, and post-test speeds of 5, 2, and 10 mm/s, respectively (Jha et al., 2006; Jha et al., 2010). During the compression of mango fruit by the cylinder probe, firmness was determined by the highest force recorded in the force-time curve recorded by the Texture Analyzer. The first peak in the texture curve was taken as peel firmness. The average force between the first and second anchors was used to calculate the flesh firmness. Fruit color and firmness concerning individual progenies were observed under three replications having a minimum of three fruits per replication. The data on various parameters were subjected for Qstats analysis to know the basic quantitative statistics, *viz*., mean, variance, standard deviation, skewness, kurtosis, and average deviation. To test the normal distribution of traits in the mapping population, a test of normality was performed, and the critical values for rejection were 5.99 and 9.21 for the tests at the 5% and 9% levels of probability, respectively.

### QTL mapping

Phenotypic data of fruit color and firmness for the bi-parental F_1_ population was used for QTL mapping with an integrated linkage map using MapQTL^®^6 (Van Ooijen, 2009; Kyazma B.V.R, Wageningen, The Netherlands) using cross-pollinated (CP) for population type and Multiple QTL model-based MQM mapping for association statistics with mapping step size of 1 cM and regression function. Other calculation parameters were set with MapQTL default. The QTL statistics were reported for those in which the LOD score exceeded the threshold, and LOD peaks were used for determining the position of a significant QTL on chromosomes.

## Results

### Linkage maps of the 20 mango chromosomes

A total of 3,317 markers heterozygous in female (1:1) and 1,517 heterozygous in both parents (1:2:1) were used for the construction of the female map. Similarly, 2,447 heterozygous in male (1:1) and 1,517 heterozygous in both parents (1:2:1) were used for male linkage mapping.

Finally, two high-density individual linkage maps with 3,213 and 1,781 SNPs distributed on 20 LGs in each segregation category were constructed as female and male maps, respectively (**Supplementary Table 1**).

The female map had 3,213 markers on 20 chromosomes with an average of 160.65 markers/chromosome. It covered a total map distance of 2,844.39 cM with individual chromosomes ranging from 82.41 cM (Chr 15) to 222.19 cM (Chr 3) with an average length of 142.22 cM. The average interval ranged from 0.43 cM (Chr 15) to 2.50 cM (Chr 3), with an average interval of 1.01 cM. The number of markers on each chromosome ranged from 75 (Chr 17) to 262 (Chr 4). This map is highly dense, and the density of SNPs ranged from 0.40 per cM (Chr 3) to 2.34 per cM (Chr 15) with an average of 1.21 SNPs per cM (**Table 3**).

**Table 3:** Genetic linkage mapping statistics.

Chr	Female map (Amrapali)			Male map (Sensation)			Integrated map		
	SNPs	Map length (cM)	Interval (cM)	SNPs	Map length (cM)	Interval (cM)	SNPs and distribute the width of columns equally	Map length (cM)	Interval (cM)
1	114	212.66	1.87	86	184.84	2.15	175	195.24	1.12
2	246	125.62	0.51	64	132.18	2.07	277	171.58	0.62
3	89	222.19	2.50	82	137.44	1.68	159	211.41	1.33
4	262	216.89	0.83	46	136.39	2.96	297	218.29	0.73
5	143	117.83	0.82	91	121.15	1.33	212	160.07	0.76
6	184	143.22	0.78	51	187.86	3.68	226	144.08	0.64
7	170	172.16	1.01	42	95.19	2.27	184	182.42	0.99
8	136	181.84	1.34	86	149.64	1.74	208	136.36	0.66
9	165	95.29	0.58	82	122.16	1.49	213	164.75	0.77
10	202	145.34	0.72	106	161.44	1.52	243	157.65	0.65
11	229	123.34	0.54	208	137.09	0.66	315	130.09	0.41
12	150	161.34	1.08	162	160.92	0.99	278	115.97	0.42
13	213	146.52	0.69	110	158.14	1.44	275	166.54	0.61
14	144	145.68	1.01	97	165.57	1.71	212	148.16	0.70
15	193	82.41	0.43	98	62.55	0.64	254	85.78	0.34
16	184	102.76	0.56	65	114.52	1.76	235	111.71	0.48
17	75	93.55	1.25	43	88.76	2.06	108	111.97	1.04
18	140	116.05	0.83	92	137.97	1.50	198	134.29	0.68
19	88	128.22	1.46	115	104.89	0.91	182	116.04	0.64
20	86	111.45	1.30	55	125.52	2.28	110	120.33	1.09
Total	3,213	2,844.39		1,781	2,684.22		4,361	2,982.75	
Mean	160.65	142.22	1.01	89.05	134.21	1.74	218.05	149.14	0.73

A total of 1,781 markers were successfully mapped on the 20 chromosomes in the male map with an average of 89.05 markers/chromosome. The male map covered a total map distance of 2,684.22 cM. The individual chromosome length ranged from 62.55 cM (Chr 15) to 187.86 cM (Chr 6), with an average length of 134.21 cM. The number of markers ranged from 42 (Chr 7) to 208 (Chr 11). The average interval ranged from 0.64 cM (Chr 15) to 3.68 cM (Chr 6), with an average of 1.74 cM. The number of SNPs per cM ranged from 0.27 (Chr 6) to 1.57 (Chr 15), with an average of 0.69 markers per cM (**Table 3**).

**Figure 2:**
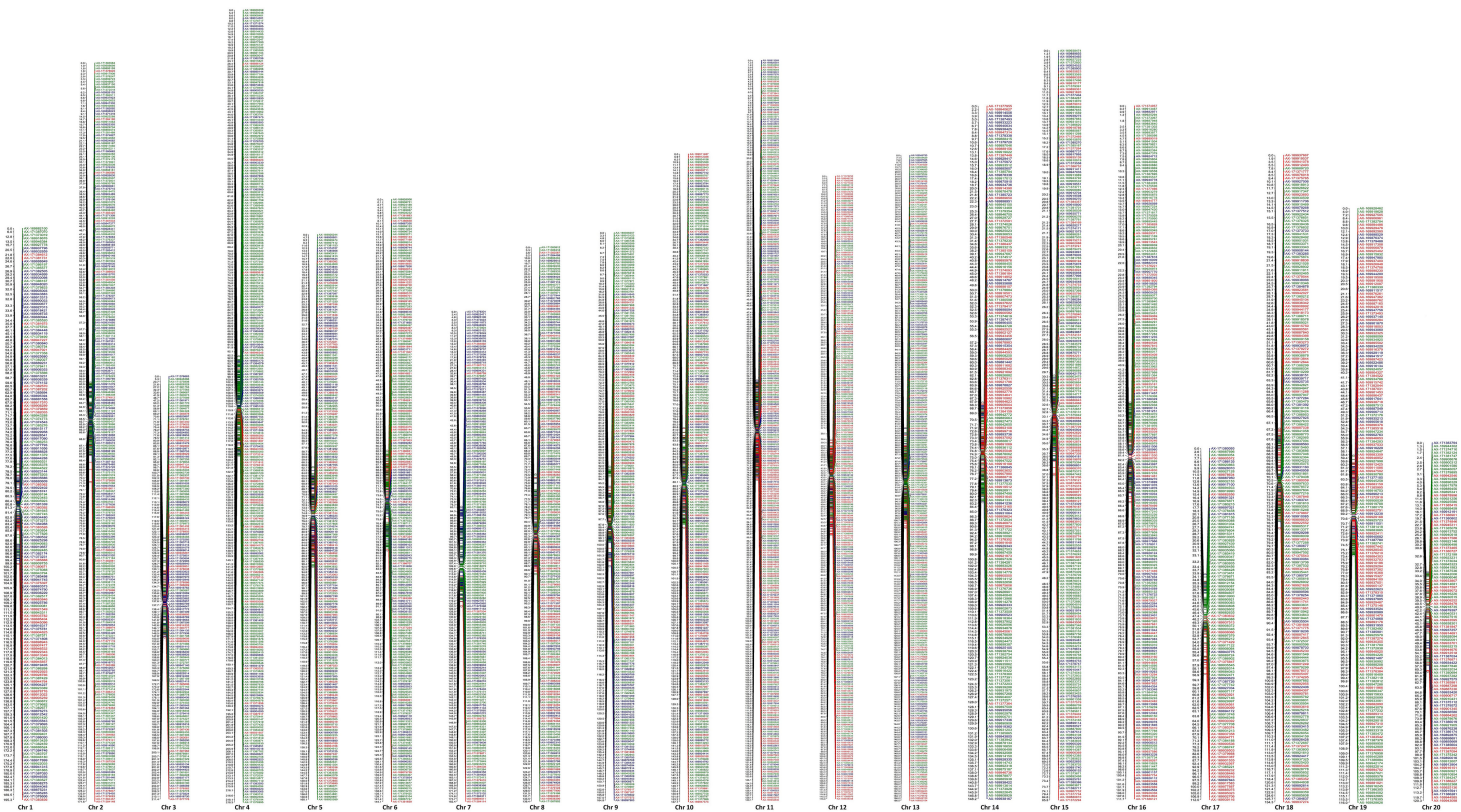
Integrated genetic linkage map and distribution of SNP markers on 20 chromosome, green color indicates marker segregating in 1:1 (Amrapali), red in 1:1 (Sensation), and blue in 1:2:1 (Amrapali/ Sensation).

Map integration was attempted using individual female and male linkage data, and a high-resolution integrated map comprising 4,361 markers mapped on the 20 chromosomes was constructed. Before the construction of integrated map, the LGs of individual maps were matched chromosome-wise, and a minimum of two markers common to individual maps were used for the construction of the integrated map. This map is highly dense, as the number of SNPs ranged from 108 (Chr 17) to 315 (Chr 11) with an average of 218.05 SNPs per chromosome. The integrated map covered a higher total map distance of 2,982.75 cM of mango genome compared to individual female and male maps. The individual chromosomes ranged from 85.78 cM (Chr 15) to 218.29 cM (Chr 4), with an average of 149.14 cM per chromosome. The average map interval on 20 chromosomes ranged from 0.34 cM (Chr 15) to 1.33 cM (Chr 3), with an average interval of 0.73 cM. The number of SNP markers per cM ranged from 0.75 for Chr 3 to 2.96 for Chr 15, with an average of 1.54 (**Table 3**; **Figure 2**).

### QTLs for the fruit quality traits

**Figure 3:**
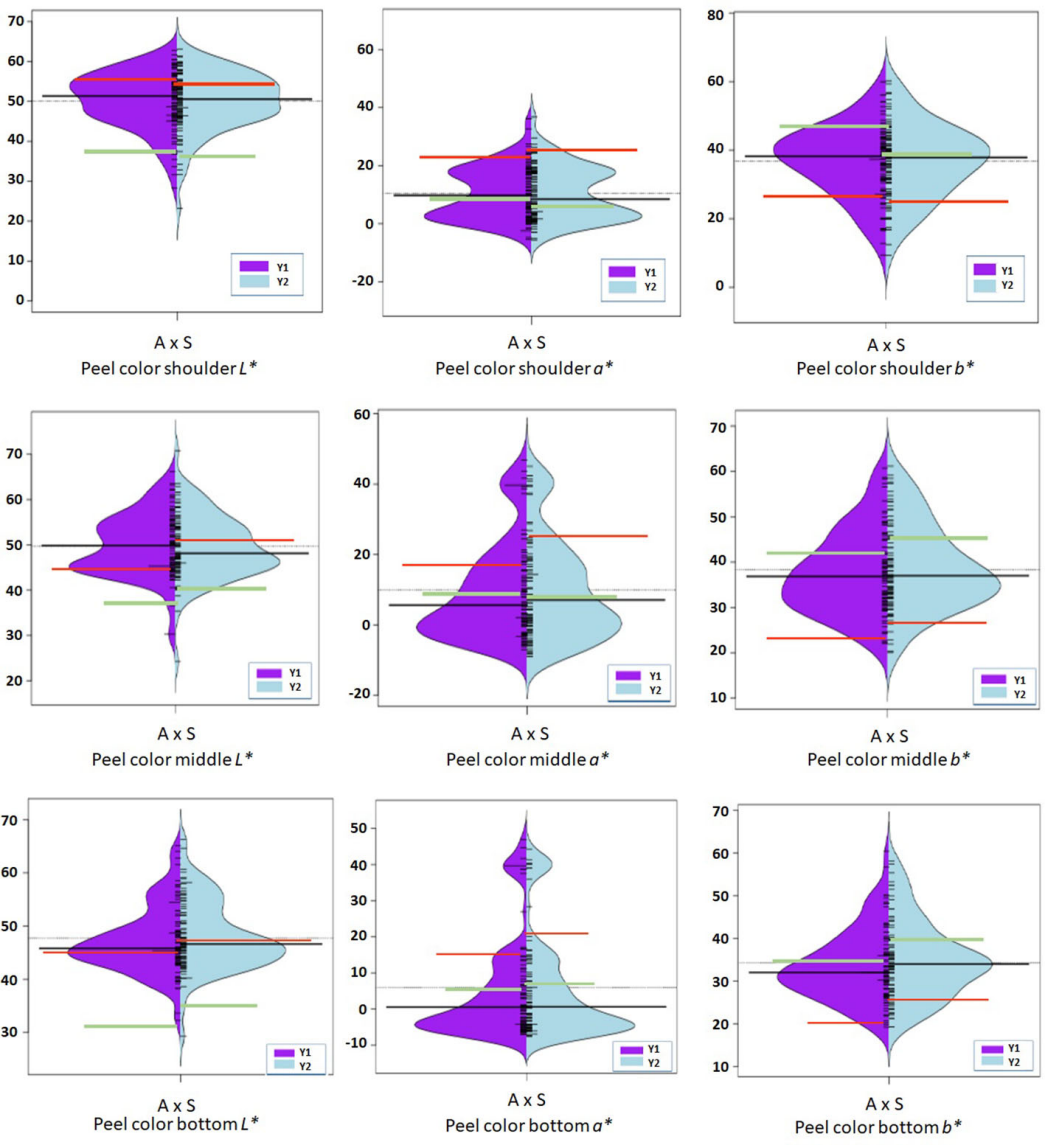
Violin-plot distribution and phenotype individual values (bars on Y axis) of peel color observed at shoulder, middle, and bottom portion of fruit in two years. Black bar median values. Green bar indicates ‘Amrapali’ female, and red bar indicates ‘Sensation’ male parental values. Symbol * used as standard symbol for chromaticity coordinates *L*, *a*, and *b*.

**Figure 4:**
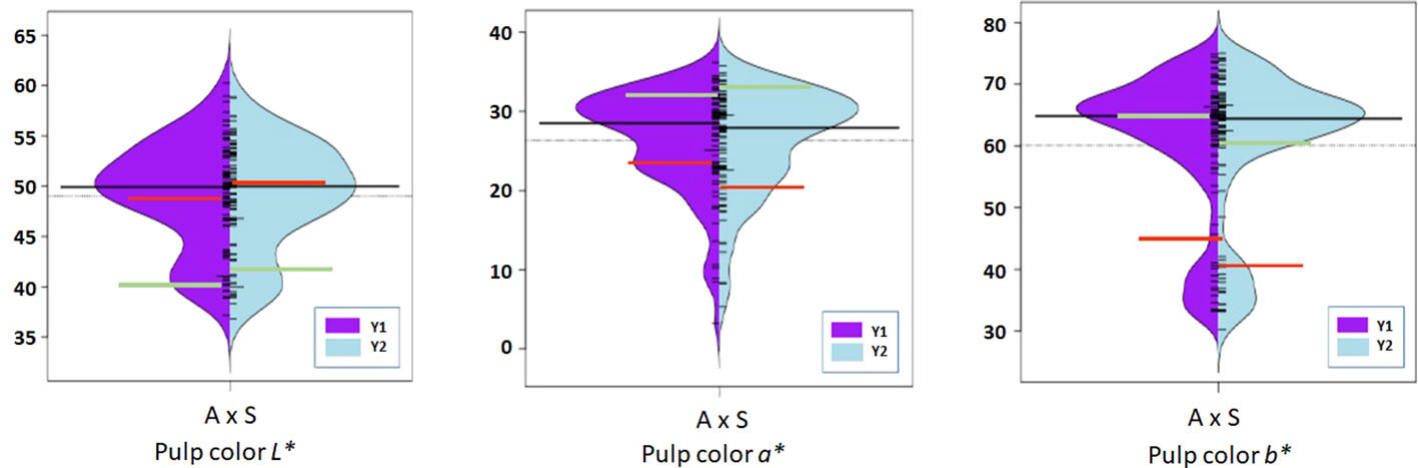
Violin-plot distribution and phenotype individual values (bars on Y axis) of fruit pulp color in two years. Black bar median values. Green bar indicates ‘Amrapali’ female and red bar indicates ‘Sensation’ male parental values. Symbol * used as standard symbol for chromaticity coordinates *L*, *a*, and *b*.

**Figure 5:**
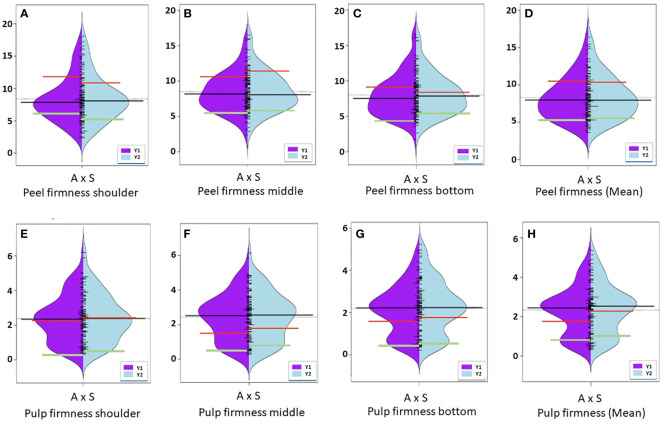
Violin-plot distribution and phenotype individual values (bars on Y axis) of peel firmness **(A–D)** and pulp firmness **(E–H)** measured at shoulder, middle, and bottom portion of fruit in two years. Black bar median values. Green bar indicates ‘Amrapali’ female, and red bar indicates ‘Sensation’ male parental values.

Phenotypic data on fruit color and firmness traits were generated for the 92 bi-parental F_1_ population for two consecutive years in 2019 and 2020 (**Figures 3**–**5**), and their mean value with genetic linkages observed in the integrated map was used for QTL mapping. Color coordinates a^*^, b^*^, and L^*^ indicate the red and yellow colors, and brightness on three points on the mango fruit in 2019, 2020, and the mean of two seasons were considered as individual traits. MQM mapping using MapQTL6 resulted in the identification of QTLs for 12 out of 20 phenotypic parameters analyzed for mapping with significant LOD scores in at least one season. **Tables 4**–**6** show the 12 traits with significant LOD scores and their QTL position on the chromosomes.

**Table 4:** Significant QTL(s) identified for mango peel color (a*, b*, L*) using integrated map data.

Season	Chr	Position (cM)	Peaks	LOD	% Expl.
a* fruit shoulder					
2019	3	49.18	AX-171381971	6.61	29.2
2020	3	49.18	AX-171381971	6.31	28.2
Mean	3	49.18	AX-171381971	6.72	28.8
2019	3	73.79	AX-171379053	8.08	34.5
2020	3	73.79	AX-171379053	7.84	34.3
Mean	3	73.79	AX-171379053	7.98	33.2
2019	3	98.75	AX-171375294	5.59	25.4
2020	3	98.75	AX-171375294	5.43	25.2
Mean	3	98.75	AX-171375294	5.58	24.6
2019	3	129.83	AX-169929951	5.73	25.9
2020	3	129.83	AX-169929951	5.13	24.0
Mean	3	129.83	AX-169929951	5.63	24.8
a* fruit middle					
2019	2	8.10	AX-171382092	4.21	19.8
Mean	12	3.40	AX-171383356	4.06	18.6
2020	17	8.64	AX-171386135	4.03	19.4
Mean	17	8.64	AX-171386135	3.98	18.2
b* fruit bottom					
2019	2	85.75	AX-169902987	4.28	20.1
2019	14	77.15	AX-169888716	4.33	20.3
2020	15	28.75	AX-169875771	4.34	20.7
Mean	15	28.75	AX-169875771	4.21	18.3
2019	18	79.42	AX-171377288	5.56	25.2
2020	18	79.42	AX-171377288	4.14	19.9
Mean	18	79.42	AX-171377288	4.92	22.0
2019	18	83.20	AX-169935441	4.63	21.5
2020	18	83.20	AX-169935441	4.40	21.0
Mean	18	83.20	AX-169935441	4.89	21.9
L* fruit shoulder					
2019	4	123.72	AX-171379971	4.73	21.9
2020	4	123.72	AX-171379971	4.68	20.2
Mean	4	123.72	AX-171379971	4.33	19.7
2019	10	46.25	AX-169943716	4.84	22.4
2019	10	76.62	AX-171376164	4.62	21.5
2020	10	76.34	AX-171378162	4.56	20.6
Mean	10	76.23	AX-171375190	4.46	20.2
Mean	10	76.62	AX-171376164	4.93	22.1
2020	15	49.25	AX-169946547	4.57	20.6
Mean	15	49.25	AX-169946547	4.57	20.6
L* fruit middle					
2019	2	33.08	AX-171373991	4.44	20.7
2020	2	33.52	AX-169925508	4.47	21.3
Mean	2	33.08	AX-171373991	4.46	20.2
2019	3	75.36	AX-171376560	4.31	20.2
2019	4	123.72	AX-171379971	4.44	20.7
Mean	4	123.72	AX-171379971	4.41	20.0
2020	15	49.25	AX-169946547	4.78	22.6
Mean	15	49.25	AX-169946547	4.39	19.9
2020	17	29.23	AX-169910005	4.77	22.6
2020	17	30.57	AX-171383828	4.34	20.7

**Table 5:** Significant QTL(s) identified for mango peel firmness using integrated map data.

Season	Chr	Position (cM)	Peaks	LOD	% Expl.
Fruit shoulder					
2020	6	89.28	AX-171383358	3.72	18.1
Mean	11	27.78	AX-169943051	3.6	16.7
2019	11	29.49	AX-169928966	3.84	18.2
2020	11	29.49	AX-169928966	3.55	17.3
Mean	11	29.49	AX-169928966	4.13	18.9
2019	11	29.64	AX-169902238	3.70	17.6
Mean	11	29.64	AX-169902238	3.98	18.2
Mean	11	30.49	AX-171382227	3.51	16.3
2020	11	30.77	AX-171380495	3.56	17.4
Mean	11	30.77	AX-171380495	3.76	17.3
2019	11	30.79	AX-169900539	3.50	16.7
2020	11	30.79	AX-169900539	3.57	17.4
Mean	11	30.79	AX-169900539	3.76	17.3
Mean	11	31.81	AX-169901747	3.75	17.3
2019	11	32.33	AX-169938965	3.76	17.9
2020	11	32.33	AX-169938965	3.70	18.0
Mean	11	32.33	AX-169938965	4.03	18.4
2020	11	32.58	AX-169925476	3.73	18.1
Mean	11	32.58	AX-169925476	3.60	16.6
2020	11	94.82	AX-169878695	4.25	20.4
Mean	11	94.82	AX-169878695	3.75	17.3
2020	11	94.97	AX-169881574	4.03	19.4
2020	11	95.19	AX-171386999	3.69	17.9
2019	20	18.15	AX-171386901	4.17	19.6
2020	20	18.15	AX-171386901	4.08	19.6
Mean	20	18.15	AX-171386901	4.28	20.2
Fruit bottom					
Mean	6	19.23	AX-169909744	3.59	16.6
2019	11	8.69	AX-169936159	3.51	16.8
Mean	11	8.69	AX-169936159	3.69	17.0
2020	11	8.69	AX-169936159	3.89	18.8
2019	11	9.16	AX-169915899	3.85	18.2
Mean	11	9.16	AX-169915899	3.96	18.2
2020	11	9.16	AX-169915899	3.64	17.7
Mean	11	17.91	AX-169900237	3.59	16.6
Mean	11	21.29	AX-169937148	3.58	16.6
Mean	11	27.78	AX-169943051	3.62	16.7
2019	11	29.68	AX-169924307	4.18	19.7
Mean	11	29.68	AX-169924307	4.83	21.7
2020	11	29.68	AX-169924307	4.79	22.6
2020	11	60.95	AX-169896842	4.08	19.6
2020	11	94.82	AX-169878695	3.73	18.1
2019	20	18.15	AX-171386901	5.06	23.3
2020	20	18.15	AX-171386901	5.25	24.5
Mean	20	18.15	AX-171386901	5.46	24.1
Average of three positions (shoulder, middle, and bottom)					
2020	11	8.69	AX-169936159	3.87	18.7
Mean	11	8.69	AX-169936159	3.51	16.3
2019	11	9.16	AX-169915899	3.58	17.1
2020	11	9.16	AX-169915899	3.85	18.6
Mean	11	9.16	AX-169915899	3.89	17.9
Mean	11	21.29	AX-169937148	3.50	16.2
Mean	11	27.78	AX-169943051	3.43	15.9
2019	11	29.68	AX-169924307	3.85	18.2
2020	11	29.68	AX-169924307	4.17	20.0
Mean	11	29.68	AX-169924307	4.30	19.6
2019	11	94.82	AX-169878695	3.58	17.1
2020	11	94.82	AX-169878695	4.28	20.5
Mean	11	94.82	AX-169878695	3.81	17.5
2020	11	94.97	AX-169881574	4.02	19.4
2019	20	18.15	AX-171386901	4.51	21.0
2020	20	18.15	AX-171386901	4.49	21.4
Mean	20	18.15	AX-171386901	4.71	21.2

**Table 6:** Significant QTL(s) identified for mango pulp firmness using integrated map data.

Season	Chr	Position (cM)	Peaks	LOD	% Expl.
Fruit shoulder					
2019	11	94.82	AX-169878695	5.08	23.3
2020	11	94.82	AX-169878695	5.80	26.7
Mean	11	94.82	AX-169878695	5.59	24.6
2019	11	94.97	AX-169881574	4.62	21.5
2020	11	94.97	AX-169881574	4.99	23.4
Mean	11	94.97	AX-169881574	4.89	21.9
Fruit middle					
2019	3	99.63	AX-169945503	4.54	21.1
2020	3	99.63	AX-169945503	4.93	23.2
Mean	3	99.63	AX-169945503	4.61	20.8
2019	11	94.82	AX-169878695	7.36	32.0
2020	11	94.82	AX-169878695	7.33	32.4
Mean	11	94.82	AX-169878695	7.42	31.3
2019	11	94.97	AX-169881574	6.78	29.9
2020	11	94.97	AX-169881574	6.53	29.5
Mean	11	94.97	AX-169881574	6.64	28.5
2019	11	95.19	AX-171386999	5.75	26.0
2020	11	95.19	AX-171386999	5.46	25.3
Mean	11	95.19	AX-171386999	5.55	24.5
Fruit bottom					
2020	4	61.59	AX-169919574	4.55	21.6
Mean	4	61.59	AX-169919574	4.79	21.5
Mean	11	21.29	AX-169937148	4.59	20.7
2019	11	94.82	AX-169878695	6.99	30.6
2020	11	94.82	AX-169878695	6.91	30.9
Mean	11	94.82	AX-169878695	7.07	30.1
2019	11	94.97	AX-169881574	6.60	29.2
2020	11	94.97	AX-169881574	6.42	29.1
Mean	11	94.97	AX-169881574	6.50	28.0
2019	11	95.19	AX-171386999	5.61	25.5
2020	11	95.19	AX-171386999	5.53	25.6
Mean	11	95.19	AX-171386999	5.50	24.3
2019	19	54.33	AX-171385518	5.14	23.6
2020	19	54.33	AX-171385518	5.33	24.8
Mean	19	54.33	AX-171385518	5.19	23.1
2020	19	55.04	AX-169902763	4.55	21.6
Mean	19	55.04	AX-169902763	4.50	20.4
2020	19	56.33	AX-171384203	4.63	22.0
Mean	19	56.33	AX-171384203	4.61	20.8
2019	19	56.47	AX-169937533	4.55	21.2
2020	19	56.47	AX-169937533	4.80	22.6
Mean	19	56.47	AX-169937533	4.72	21.3
Average of three positions (shoulder, middle, and bottom)					
Mean	11	21.29	AX-169937148	4.63	20.9
2019	11	94.82	AX-169878695	6.97	30.6
2020	11	94.82	AX-169878695	7.48	33.0
Mean	11	94.82	AX-169878695	7.28	30.8
2019	11	94.97	AX-169881574	6.42	28.5
2020	11	94.97	AX-169881574	6.65	30.0
Mean	11	94.97	AX-169881574	6.49	28.0
2019	11	95.19	AX-171386999	5.47	24.9
2020	11	95.19	AX-171386999	5.61	26.0
Mean	11	95.19	AX-171386999	5.46	24.1
2020	19	54.33	AX-171385518	4.71	22.3

#### Peel and pulp color

**Figure 6:**
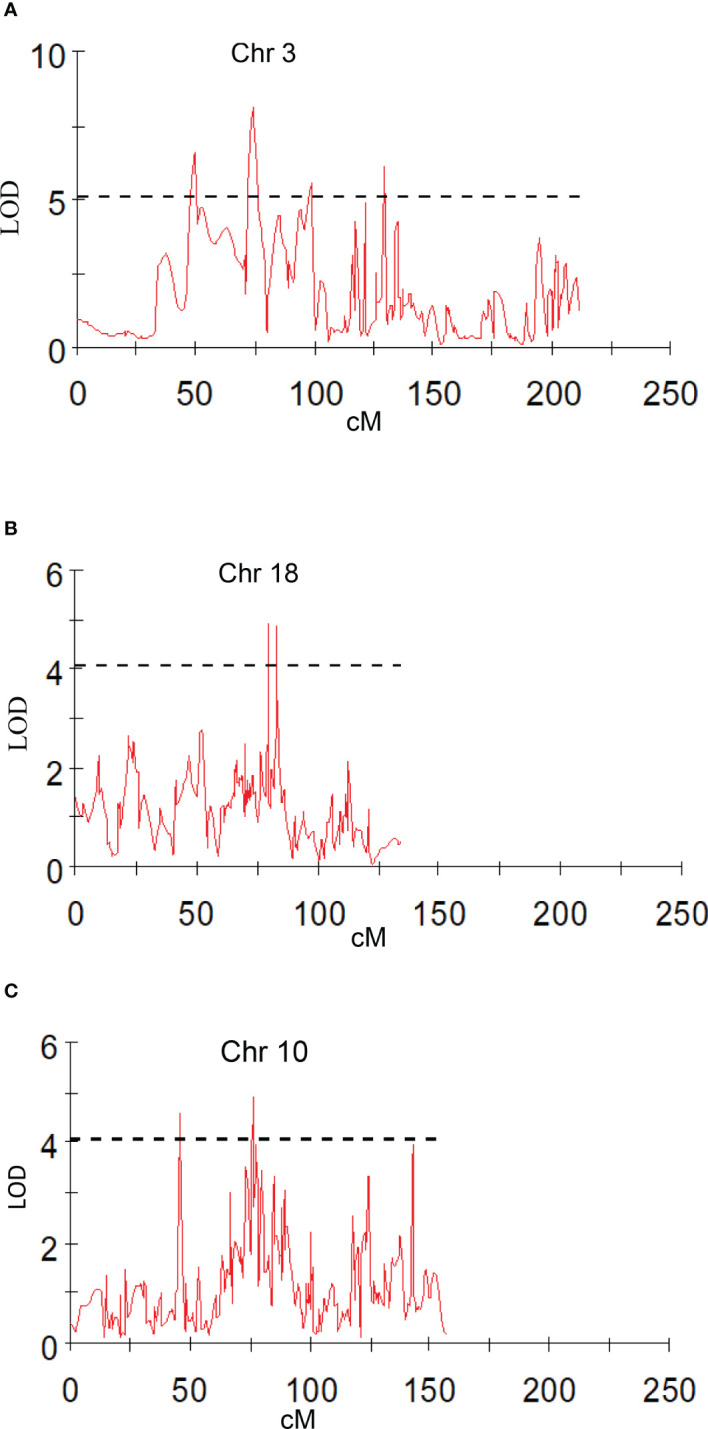
Graphs of the plot of the likelihood of the odds that a SNP marker is associated with the trait. **(A)** peel a* shoulder of fruit; **(B)** peel b* bottom of fruit; **(C)** peel L* shoulder of fruit in 2019.

Chromaticity coordinates observed on the shoulder, middle, and bottom portion of mango fruits revealed that expression of a* indicative of red color on the shoulder is associated with Chr 3 of integrated genetic linkage map. Four significant QTLs were identified, one at a position of 49.18 cM (6.31 to 6.72 LOD) explains 28.2 to 29.2% of phenotypic variation, the second at a position of 73.79 cM (LOD 7.84 to 8.08) explaining 33.2 to 34.5% of phenotypic variance, the third QTL at a position of 98.75 cM (5.43 to 5.59 LOD) and fourth at 129.83 cM (LOD 5.13 to 5.73) explain around 25.0% of phenotypic variation in the population. These QTLs were observed in both seasons, i.e., 2019 and 2020, as well as with the mean phenotypic values over the years (**Table 4**; **Figure 6A**). However, expression of a* at middle of fruit is associated with Chr 2, 12, and 17. In the present study, peel a* value observed at fruit bottom did not result in the identification of any significant QTLs. This may be because the observed red blush on the shoulder differed significantly compared to the middle and bottom portions of the fruit. Expression of b* presenting yellow color is associated with Chr 2, 14, 15, and 18. Results revealed that Chr 2 showed one QTL at a position of 85.75 cM (LOD 4.28; R2 20.1), and Chr 14 showed another QTL at a position of 77.15 cM, explaining 20.3% phenotypic variation in 2019. Similarly, Chr 15 showed one QTL in 2020 with mean value at a position of 28.75 cM and with a LOD score of 4.21 to 4.34, explaining 18.3 to 20.7% phenotypic variations in the population (**Table 4**). Two consistent QTLs identified on Chr 18 (79.42 and 83.20 cM) explain 19.9 to 25.2% phenotypic variations for b* of fruit bottom (LOD 4.14-5.56) (**Figure 6B**). It was also noted that the b* value observed at the fruit shoulder and middle did not yield any significant QTL. QTLs governing the brightness of fruit were located on Chr 2, 3, 4, 10, 15, and 17 (**Table 4**; **Figure 6C**). We did not observe any significant QTL(s) for pulp color in the present study.

#### Peel and pulp firmness

**Figure 7:**
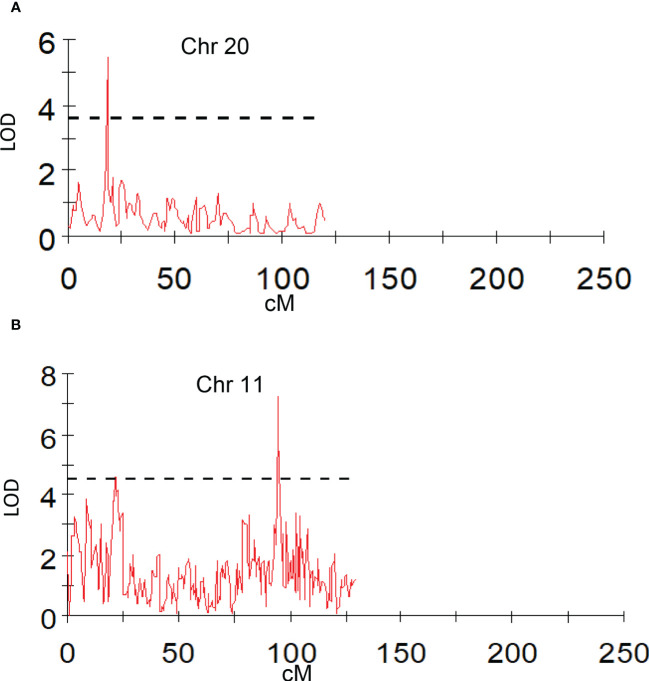
Graphs of the plot of the likelihood of the odds that a SNP marker is associated with the trait. **(A)** peel firmness observed at fruit bottom; **(B)** pulp firmness at fruit shoulder (mean of 2019 and 2020).

Peel firmness observed at shoulder and bottom of mango fruit was associated with SNPs located on Chr 6, 11, and 20. Total 12 SNPs on Chr 11 at position ranging from 27.78 to 95.19 cM (R2 16.3-20.4) were identified as having an association with peel firmness at fruit shoulder. Similarly, eight SNPs hosted on Chr 11 at a position 8.69 to 94.82 cM (LOD 3.51 to 4.83; R2 16.6-22.6) consistently appeared with traits observed on fruit bottom (**Table 5**). One QTL at a position of 18.15 cM (LOD 4.08-5.46; R2 19.6-24.5) on Chr 20 appeared consistently, with peel firmness observed on fruit shoulder and bottom in both seasons (**Figure 7A**). One minor QTL at Chr 6 at 19.23 cM (LOD 3.59; R2 16.6) was also identified. Fruit firmness measured at the fruit middle did not result in significant QTLs, while firmness observed at the shoulder and bottom of the fruit confirmed significant SNP association. However, average peel firmness of three different positions confirmed association of seven SNPs.

SNPs located on Chr 3, 4, 11, and 19 had an association with pulp firmness (**Table 6**). SNPs located on Chr 11 at a position of 94.82 to 95.19 cM (LOD 4.54-7.42; R2 20.4-32.4) consistently appeared for pulp firmness observed at three different positions on the fruit and with the mean value (**Figure 7B**). One QTL on Chr 3 at a position of 99.63 cM (LOD 4.54-4.93), explaining 20.8-23.2% of phenotypic variations, was also identified. Pulp firmness observed at the fruit bottom showed an additional four SNPs on Chr 19 at a position of 54.33-56.47 cM (LOD 4.50-5.33) and explains 20.4-24.8% of phenotypic variations in the population. QTL analysis using a year-wise mean of pulp firmness observed at different positions of mango fruits confirmed three SNPs on Chr 11 (94.82 to 95.19 cM; LOD 5.47-7.48; R2 24.1-33.0).

## Discussion

### High density genetic maps from SNP markers

Traditional mango breeding is cumbersome and time-consuming. In the present decade, rapid advancement in DNA sequencing and molecular genetic techniques has generated a wealth of information on mango genomics. Apart from conventional breeding approaches, efforts have been made in the recent past to utilize biotechnological tools for marker-aided breeding. Significant progress has been made in the area of genome sequencing of mango by several researchers (Singh et al., 2014; Luo et al., 2016; Singh et al., 2016; Kuhn et al., 2017; Wang et al., 2020). The wealth of genome resources has also been generated by our group as genome sequences of important mango cultivars (unpublished), and millions of SNPs have been identified, which has immense value in future mango breeding (Singh et al., 2014; Mahato et al., 2016; Singh et al., 2016; Iquebal et al., 2017).

As a key step in genetic linkage mapping, the mapping population is highly important. F_1_, F_2_, backcross populations, and haploid populations can be used for genetic mapping (Wang, 2001). The F_1_ population is an ideal mapping population for highly heterozygous tree species like mango and can be obtained by one-generation hybridization between contrasting parents with high heterozygosity (Lu et al., 2019). There are several examples where genetic maps have been constructed based on the F_1_ population such as *Poncirus trifoliata* (Xu et al., 2021), *Ziziphus jujuba* (Wang et al., 2019), *Persea americana* (Rendón-Anaya et al., 2019), *Vitis* (Zhu et al., 2018), *Coryus avellana* (Bhattarai and Mehlenbacher, 2017), and *Theobroma cocoa* (Argout et al., 2011). We chose to generate genetic linkage map using bi-parental F_1_ population derived by crossing Amrapali/Sensation contrasting for fruit quality traits. Full-sib populations from two known parents are considered more effective for breeding progress than half-sib populations from open pollinated maternal parents. Genetic maps that are based on segregating full-sib hybrid populations are better for establishing linkages between horticultural traits and molecular markers for MAS (Kuhn et al., 2017).

Moreover, SNP marker-based genetic maps have several advantages, as SNPs are more abundant, easier to identify, easier to score, and unambiguous markers (Kuhn et al., 2017). Studies have shown that SNP loci are ubiquitous in the genome and the most abundant forms of genetic variation between individuals of the same species (Rafalski 2002; Kuhn et al., 2017). In this study, a mango 80K genic-SNP genotyping array was used to genotype the F_1_ full-sib (Amrapali/Sensation) population along with parents.

High-density genetic maps are valuable in genetic and genomic studies, illuminating the genetic and molecular mechanisms of plants and providing the necessary framework for QTL analyses, gene cloning, and molecular breeding (Wang et al., 2019; Wu et al., 2019). We used 4,834 and 3,964 SNPs for construction of female and male maps. However, not all the SNPs that expected to segregate in a disomic fashion were able to be assigned to a linkage group. In female, 3,213 SNPs and in male 1,781 SNPs could be successfully mapped on 20 LGs. As the mango has 40 chromosomes with the haploid number of 20, we were successful in identifying 20 LGs for both female and male maps. This suggests the diploid nature of mango, and even though it is a partial allopolyploid, the two ancestral genomes were different enough to be distinguished by SNP markers (Kuhn et al., 2017). Genetic linkage data of female and male maps were used for integration, and a high-density linkage map was constructed, having 4,361 SNPs that covered the entire genome of mango. The map length covered by female, male, and integrated maps slightly differed for number of SNPs. The 3,213 SNPs of the female map covered 2,844.4 cM of the genome while 1,781 SNPs of the male map covered 2,684.2 cM. Integration of female and male maps resulted in 4,361 SNPs distribution across 20 chromosomes covering 2,982.8 cM of map length.

Few genetic maps in *Mangifera indica* have been constructed using different markers (Kashkush et al., 2001; Singh et al., 2014; Luo et al., 2016; Singh et al., 2016; Kuhn et al., 2017; Wang et al., 2020). The integrated genetic linkage map reported here is of high density, as the number of markers is reasonably high. In this study, we used a strategy to make the map that took advantage of the strengths of markers segregating differently. Further, markers corresponding to the same chromosome from all segregation groups were integrated. Our genetic map is significantly better than previous maps, as it is highly dense, based on full-sib (Amrapali/Sensation) population, and covered the entire mango genome.

### Trait association to the genetic maps

Marker assisted selection provides ample opportunity to reduce the mango breeding cycle and improve efficiency as well. In recent decade, the advancements made in the augmentation of genome resources in mango (Kashkush et al., 2001; Luo et al., 2016; Singh et al., 2016; Kuhn et al., 2017; Wang et al., 2020) provide unprecedented opportunities to breeders for MAS in mango. Mango fruit is adored by people for its taste and nutrition, contributed by color, flavor, and aroma. Among these, peel and pulp color are important traits contributing to fruit quality and market value (Bajpai et al., 2017). Fruit firmness is another important trait for storage, transportation, and disease and insect management. Mapping populations from controlled crosses are not easy to generate in mango due to the high level of technical proficiency required (Bally et al., 2021). The F_1_ bi-parental progeny population studied is few in fruit trees compared to annual crops (Grattapaglia and Sederoff, 1994). In the present study, we attempted to elucidate the regions of mango genome influencing fruit color and firmness using a F_1_ bi-parental (Amrapali/Sensation) mapping population. To be useful for marker-aided breeding, it is imperative to have markers showing strong association with the horticultural traits. A map is not necessary to identify markers associated with a trait, but confidence in this association increases as multiple markers near the trait locus on the genetic map also show significant association with the trait (Kuhn et al., 2017). Fruit quality-related traits are quantitative traits that are influenced by multiple genes, and perhaps no single gene shows a significant impact on a trait. In our study, MQM mapping was used. This method has also been used in QTL analyses for quality traits in *Gossypium hirsutum* (Zhang et al., 2019), *Poncirus trifoliata* (Xu et al., 2021), and *Elymus sibiricus* (Zhang et al., 2019). In this study, we could identify SNP association for 12 of 20 traits used for analysis.

### Peel and pulp color

Fruit color is a highly variable trait within the fruit and is much influenced by environmental conditions. Precise qualitative evaluation of peel color is influenced by the number of fruits examined and from which part of the tree it is collected. More randomly chosen fruits from all parts of a tree may reduce this variation. In addition, we used another approach for determining the peel color using the Hunter color meter and took multiple observations on different positions of the fruit. This approach provided us with quantifiable data more suitable for QTL mapping compared to data based on scales or grouping. It was evident that red blush was more towards the shoulder compared to the middle and bottom portions of the fruit. An attractive fruit color is one of the most important factors for export markets (Nambi et al., 2016). The accumulation of pigments and their concentration and intensity determine the overall appearance of color in mango fruits (Pervaiz et al., 2017). Red coloration in fruits and other plant tissues has multifarious roles such as conferring plant disease resistance and protection against UV radiation (Bieza and Lois, 2001; Berardini et al., 2005; Sivankalyani et al., 2016). Anthocyanins also provide human health benefits against cancer and cardiovascular and other chronic diseases (Rao and Rao, 2007; Butelli et al., 2008; Singh et al., 2008). We found that fruit peel a* indicating red blush on the shoulder was associated with Chr 3. Four QTLs (AX-171381971, AX-171379053, AX-171375294, and AX-169929951) with high LOD value of 5.13-8.08 explaining around 35% of phenotypic variation were identified. The a* value observed on the middle of fruit resulted in associated SNPs located on Chr 2, 12, and 17. However, a* observed on the bottom portion of the fruit did not result in any QTL(s) in the present study. This may be because the appearance of red blush is more significant on the shoulder compared to the middle and bottom portion of the fruits. The SNPs associated with peel yellow color (b*) trait was on Chr 2, 14, 15, and 18. Mango fruits are classified based on peel color into green, yellow, and red types. Mango peel turns from green to yellow or red or retains green colors during ripening. Carotenoids and anthocyanins are the important pigments responsible for the colors of fruits. Pigments in different proportions may have an influence on the expression of different shades of color on mango peel. The genetics of peel color has not been studied in detail, but available reports indicate that it is governed by several genes and regulated in a more complex manner. In agreement with this, we observed several SNP markers housed on different chromosomes that had an influence on peel color development in mango. Consistency of these QTLs over phenotypic values observed in different seasons confirms their involvement in the expression of fruit color.

### Peel and pulp firmness

Mango varieties differ considerably for fruit peel and pulp firmness. Fruit firmness is a significant quality aspect of mango for consumers, as it represents ripeness and influences shelf-life, transportation, and processing issues (Jha et al., 2010). Various industries use puncture tests as part of their quality control procedure. Cool store operators monitor firmness throughout the storage period as part of their inventory management. In addition, firmness is sometimes used to predict consumer responses. Stec et al. (1989) found that preferred firmness at eating ripeness varied among assessors (Watkins and Harman, 1981; Harker et al., 1996). Jha et al. (2006) reported that the firmness of the mango fruits remained almost constant over the period of growth, and it decreased after attaining maturity. It was also suggested that the maturity of mango could be predicted by measuring size, color, and firmness. We used the approach of determining the peel and pulp firmness on different positions of fruits. We observed several SNPs associated with the peel and pulp firmness of mango fruits housed on Chr 3, 4, 6, 11, 19, and 20. SNPs on Chr 11 and Chr 20 consistently appeared in all seasons of observation and mean value of peel firmness. SNPs located on Chr 3, 4, 11, and 19 had an association with pulp firmness. SNPs located on Chr 11 at a position of 94.82 to 95.19 cM consistently appeared in different seasons. One QTL on Chr 3 at a position of 99.63 cM was also identified. The work reported here is unique concerning peel and pulp firmness results, where QTLs are reported for the first time in mango.

## Conclusions

We demonstrated the usefulness of designing a mapping population from two commercially important mango cultivars, Amrapali and Sensation, that have phenotypic polymorphism for fruit peel color and firmness traits. The integrated genetic recombination map using segregation data of female, male, and both parents reported here is unique and reasonably resolved. Our analysis of the association of SNPs for fruit color and firmness traits is novel and enables us to formulate mango breeding strategies that can improve breeding efficiency in identifying desirable progeny with optimal horticultural traits. The information about genetic linkages and QTLs generated would be highly useful in mango breeding and for broadening the understanding of the genetics of these traits. This knowledge will allow breeders to design trait-specific breeding strategies in mango.

## Data availability statement

The original contributions presented in the study are included in the article/**Supplementary Material**. Further inquiries can be directed to the corresponding author.

## Author contributions

MS, NKS, SKS, RS, and NSi conceptualized the project and organized the work plan for the manuscript. MS, SR, PJ, NSi, SaS, and AM contributed to genotyping. MS, SR, AG, RD, NR, SHS, GK, AN, and ShS contributed to phenotyping. MS, NR, GT, PJ, SJ, MI, RS, and PP contributed to data analysis. AK, SRG, and ShSe extended the lab facilities for phenotyping. NSh helped in manuscript writing. All authors contributed to the article and approved the submitted version.
